# Alu Elements as Novel Regulators of Gene Expression in Type 1 Diabetes Susceptibility Genes?

**DOI:** 10.3390/genes6030577

**Published:** 2015-07-13

**Authors:** Simranjeet Kaur, Flemming Pociot

**Affiliations:** 1 Department of Pediatrics, Copenhagen Diabetes Research Center (CPH-DIRECT), Herlev University Hospital, Herlev Ringvej 75, Herlev DK-2730, Denmark; E-Mail: simranjeet.kaur@regionh.dk; 2 Faculty of Health and Medical Sciences, University of Copenhagen, Copenhagen DK-1165, Denmark

**Keywords:** Alu, T1D, repeat elements, IRAlus, UTRs

## Abstract

Despite numerous studies implicating Alu repeat elements in various diseases, there is sparse information available with respect to the potential functional and biological roles of the repeat elements in Type 1 diabetes (T1D). Therefore, we performed a genome-wide sequence analysis of T1D candidate genes to identify embedded Alu elements within these genes. We observed significant enrichment of Alu elements within the T1D genes (*p*-value < 10e−16), which highlights their importance in T1D. Functional annotation of T1D genes harboring Alus revealed significant enrichment for immune-mediated processes (*p*-value < 10e−6). We also identified eight T1D genes harboring inverted Alus (IRAlus) within their 3' untranslated regions (UTRs) that are known to regulate the expression of host mRNAs by generating double stranded RNA duplexes. Our *in silico* analysis predicted the formation of duplex structures by IRAlus within the 3'UTRs of T1D genes. We propose that IRAlus might be involved in regulating the expression levels of the host T1D genes.

## 1. Introduction

Alu repeat elements are the most conspicuous human SINES (short interspersed nuclear elements), covering about 11% of the human genome with over one million copies, and are generally found in gene-rich regions [1,2]. On average, an Alu repeat (~300 nt) unit occurs approximately once every 4 kb in the genome [2]. Alus are known to be involved in various deletions and translocation junctions with pathological implications, and mispairing between two Alu elements has been shown to be the main cause of base deletions and duplications [3,4]. Alu elements frequently serve as gene enhancers and promoters or are used as sites for alternative mRNA splicing [5]. These elements have wide-ranging influences on various cellular processes including polyadenylation, gene expression, RNA editing, and translation regulation [6]. The overwhelming majority of Alu repeats are found within the intronic regions of the human genome. The embedded Alus have been found to be enriched within both 5' and 3' untranslated regions (UTRs). Particularly, antisense Alus have been shown to have positional preference within the embedded Alus, with significant enrichment in the 3'UTRs [7]. Alu elements within 3'UTRs are potential target sites for the microRNAs (miRNAs) and other small noncoding RNAs; in particular, sense Alu sequences are enriched for potential miRNA target sites [8,9,10]. Interestingly, genic Alus within chromosomes 21 and 22 have been found to be enriched in genes involved in metabolism, transport, and signaling processes [11]. A typical Alu element is a dimer composed of a central A-rich region flanked by two similar left and right arms that are related to the 7SL RNA [12]. The recent extensive expansion of Alus has resulted in the generation of a series of Alu subfamilies of different ages that can be classified based on their insertions, deletions, and mutations. The major Alu subfamilies are: Alu J (oldest), Alu S (middle), and Alu Y (youngest). The most common members of the Alu S family include AluSx, AluSg, AluSp AluSc, and AluSq, whereas the members of the Alu J family include AluJo and AluJb. In general, the Alu subfamilies have high sequence identity among themselves (70%–99.7%).

Adenosine-to-inosine (A-to-I) editing of double-stranded RNAs (dsRNAs) mediated by adenosine deaminase proteins (ADARs) is crucial for normal life and development and has been found to play roles in various human diseases [13]. Inter- or intramolecular base-pairing interactions between inverted Alus (two opposite oriented Alu elements) give rise to dsRNA structures, which are preferred substrates for ADARs. More than 90% of genome-wide A-to-I editing events are localized within inverted Alu (IRAlu) elements [14]. The presence of IRAlus within the 3'UTRs of human mRNAs has been shown to alter gene expression and translational efficiency [14]. Recently, intramolecular base-pairing of IRAlus present within 3'UTRs and long non-coding RNAs (lncRNAs) has been shown to be involved in Staufen1 (STAU1)-mediated mRNA decay (SMD) [15]. For example, RNA duplexes formed as a result of the base-pairing of either AluJo (100 nt) in the 3'UTR of *SERPINE1* or AluSx (300 nt) in the 3'UTR of *FLJ21870* with an Alu repeat located within a lncRNA *AF087999* are known targets for SMD [15]. Interestingly, Alu elements embedded within lncRNAs have also been found to be frequent templates for A-to-I editing, which further confirms that IRAlus form stable dsRNA structures [16].

Approximately 0.3% of all human genetic disorders, including forms of breast cancer and acute myelogenous leukemia, are thought to have resulted from Alu-mediated unequal homologous recombination. Inherited diseases like Type 2 diabetes, Lesch-Nyhan syndrome, Tay-Sachs disease, complement component C3 deficiency, familial hypercholesterolemia, and thalassemia have also been also associated with Alu-mediated recombination [17]. Insertion of an Alu element in *NF1* gene causes Neurofibromatosis type 1 [18]. A severe form of hemophilia occurs by an Alu element insertion into intron 18 of the human factor VIII, leading to the deletion of exon 19 during the splicing process [19].

In a recent study [20], we observed significant enrichment for Alu repeats within Type 1 diabetes (T1D) loci-associated lncRNAs compared to the background (all known lncRNAs). T1D is a multifactorial disease mostly affecting children and young adults. T1D results from the chronic immune-mediated destruction of insulin-producing β-cells of the pancreatic islets of Langerhans [21]. The cellular roles of Alu repeats in T1D remain largely unexplored. Therefore, in this study, we performed a sequence-based analysis of T1D candidate genes to explore the relative distribution of Alu repeat elements. We postulate that the embedded Alu RNAs may serve an important function in regulating the expression of T1D genes.

## 2. Materials and Methods

T1D candidate genes associated with T1D loci were retrieved from T1Dbase (v. 4.16) (www.t1dbase.org). All sequences for the T1D genes were retrieved from Ensembl v. 75 (Grch37) BioMart [22] including intronic sequences, coding sequences, and 5' and 3'UTRs. The intronic, coding, and UTR sequences were retrieved for all known isoforms. The repeat-masking process was performed on the T1D genes, T1D introns, T1D coding sequences, and T1D 5' and 3'UTRs separately. RepeatMasker open-4.0.5 [23] with rmblastn (version 2.2.27+) and RepBase (a well curated library of known repeat family consensus sequence, version 20140131) [24] were used for identifying all repeat elements. All human UTR sequences were retrieved from BioMart (Ensembl v. 75). The pre-masked human genome was retrieved from RepeatMasker (based on hg19 build, RepeatMasker open-3.3.0 and RepBase (version 20120124). All statistical analyses were performed in R programming language. Enrichment testing for specific repeat elements was performed using a chi-square test to identify statistically significant and over-represented repeat elements, based on the null hypothesis that there is no enrichment or over-representation for a specific repeat category in the selected group. We used RNAfold and RNAcofold from Vienna 2.0 package [25] for secondary structure prediction of IRAlus.

### Functional Annotation and Enrichment Analysis

The gene ontology (GO) and pathway-based annotation and enrichment analysis of T1D genes harboring Alu elements was performed using DAVID (the Database for Annotation, Visualization, and Integrated Discovery) [26]. GO analysis was performed based on GO-Fat categories. GO-Fat attempts to filter out the broadest terms to avoid overshadowing of the more specific terms. A total of 364 T1D genes were mapped with DAVID Ids out of a list of 554 T1D genes harboring Alu elements. KEGG pathway annotations were used for pathway-based analysis. We used the functional annotation module to identify top enriched GO terms and KEGG pathway annotations [27] for T1D genes harboring Alu repeats (gene count ≥ 10, EASE score <0.05, Bonferroni correction < 0.05). In DAVID, enrichment for each group is measured by the geometric mean of all EASE scores (significant or insignificant) associated with the enriched annotation terms that belong to that gene group. The EASE score (a modified Fisher’s exact *t*-test) is calculated by penalizing (removing) one gene within a given category from the list and calculating the resulting Fisher exact probability for that category, and it therefore represents the upper bound of the Fisher exact probability distribution. This is advantageous in terms of penalizing the significance of categories supported by fewer genes. Functional annotation clustering module in DAVID was used for measuring relationships among the annotation terms on the basis of the degree of their co-association with genes within the query list to cluster somewhat heterogeneous yet highly similar annotation into functionally annotated groups. The enriched clusters with group enrichment scores less than or equal to 0.05 (equivalent to 1.3 on the minus log scale) were selected. A higher group enrichment score indicates that the majority of its gene members are associated with highly enriched annotation terms and involved in more important (enriched) roles. The clusters were ordered by group enrichment scores, and the representative biological terms associated for each enriched cluster (group enrichment score above 1.3) were manually selected, providing a much clearer and non-redundant view of the annotation terms.

## 3. Results and Discussion

We screened for all repeat elements within T1D candidate genes and compared the relative abundance of various classes of interspersed repeats. We retrieved all T1D candidate genes associated with T1D susceptibility loci from T1Dbase v4.16 (www.t1dbase.org). In total, 941 T1D candidate genes were retrieved (Table 1). Sequences were retrieved for these 941 T1D candidate genes (for brevity we will refer to these as T1D genes hereafter) from Ensembl v75 (GrCh37) BioMart [22]. In addition, we also retrieved coding, intronic, and UTR sequences for these 941 T1D genes to compare the overall distribution of Alu elements within the T1D genes (Table 1). Overall, for the T1D genes, 2419 coding, 2403 intronic, 2048 5'UTR, and 1758 3'UTR sequences were retrieved, which included all reported isoforms of T1D genes. The repeat masking was performed using RepeatMasker [23] (www.repeatmasker.org) and RepBase [24]. The pre-masked human genome from RepeatMasker was used for comparison. All human UTR (both 5' and 3'UTRs) sequences were retrieved for comparison of the repeat element distribution with T1D 5' and 3'UTRs. In total, 80,296 5'UTRs and 72,248 3'UTRs sequences were retrieved for all human genes, including all isoforms.

**Table 1:** Characteristics of T1D genes. The sequence-based characteristics of T1D genes, T1D coding sequences (CDS), T1D intronic sequences, and T1D UTRs are based on their length and GC content. The CDS, introns, and UTRs include all isoforms of the T1D genes.

Characteristics	T1D genes	CDS	Introns	5'UTRs	3'UTRs
Sequences	941	2419	2403	2048	1758
Total length	20,074,601 nt	2,568,639 nt	64,787,126 nt	491,916 nt	1,287,596 nt
GC level	44.54%	55.81%	43.65%	59.87%	48.10%
Average length	10,531	1061	7435	240	732
Max length	32,759	15,018	32,753	3474	11,007

### 3.1. T1D Genes Are Enriched for Alu Elements

The interspersed repeat elements, comprised of LINES (long interspersed nuclear elements), SINEs, LTRs (long terminal repeats), and DNA elements, account for approximately 50% of the human genome. LINEs, being the most abundant repeat elements, comprise ~22% of the human genome, followed by SINEs (~13%), LTRs (~9%), DNA elements (~3%), and other repeat elements (~2%). However, in the case of T1D genes, SINEs were found to be the most abundant repeat elements (~18%), followed by LINEs (~15%), LTRs (5%), DNA elements (~3%), and other repeats (~2%) (see Supplementary Table S1).

**Figure 1:**
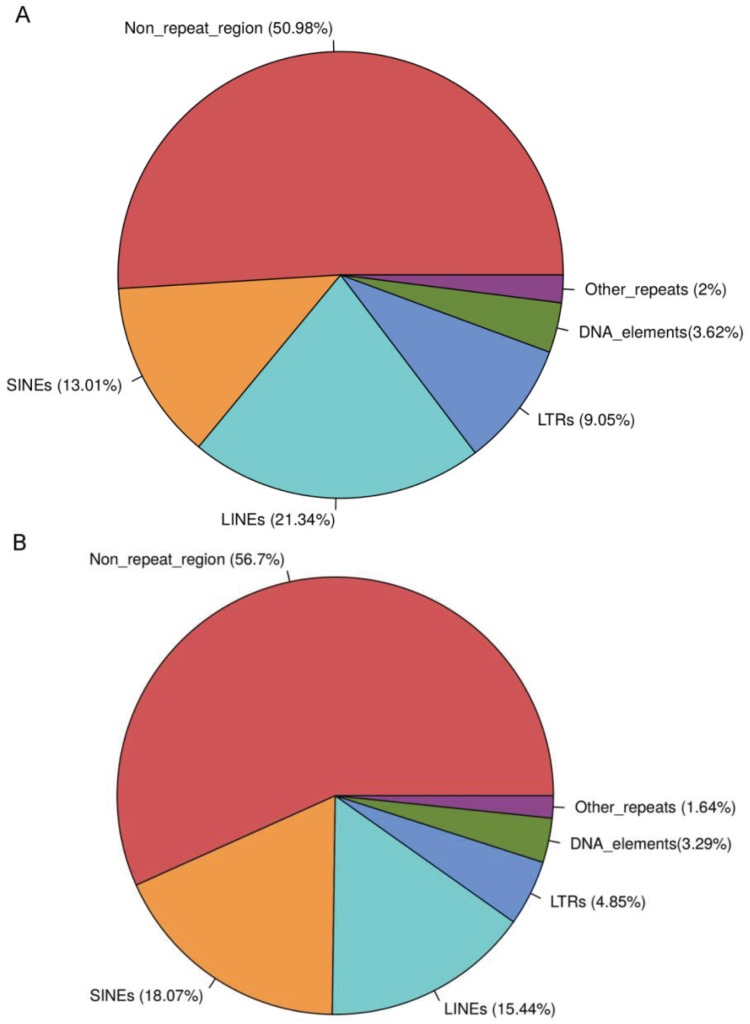
Repeat elements distribution in the human genome (**A**) and T1D genes (**B**). The figure shows the percentage sequence covered by different classes of interspersed repeat elements.

Among SINEs, Alu repeat elements are the most abundant in the human genome; therefore, we only focused on Alu elements for further analysis. We observed significant enrichment of Alus within T1D genes (*p*-value < 2.2e−16, Chi-square test) compared to the background Alu distribution in the human genome (Figure 1). In our previous study [20], we also compared the distribution of Alus in Inflammatory Bowel Disease (IBD) with T1D loci and the human genome; however, we did not observe any enrichment of Alus within IBD candidate genes.

The Alu repeats covered ~15% of the sequence in the T1D genes as compared to ~11% of the sequence in the human genome. Around 59% of the T1D genes (554 genes) harbored Alu elements (Table 2). Although the average expected frequency of Alus is one copy per 4 kb in the human genome, high density clustering of Alu repeats is known to occur in certain regions, particularly within GC-rich regions. For T1D genes, we observed a significantly higher frequency of Alus *i.e.*, one Alu per 1.8 kb. We also observed a higher GC content (44.5%) for T1D genes compared to the average GC content of the human genome (41%) (*p*-value < 2.2e−6, Welch two sample t-test). Also, in the case of T1D 5' and 3'UTRs, a higher GC content was observed as compared to the background (all human UTRs). The average GC content for T1D 3'UTRs was found to be 48%, whereas background 3'UTRs had an average GC content of 44%. In the case of T1D 5'UTRs, we observed a slightly higher GC content (59%) compared to the background 5'UTRs (58%). Since T1D genes were found to be GC rich, this observation alludes to the positional preference of Alu elements within GC-rich regions. A list of the top 20 T1D genes based on their total number of Alu elements is shown in Table 3. Interestingly, these genes enriched for Alu elements included some of the well-characterized T1D candidate genes—for example *BACH2*, *SKAP2*, *PTPN2*, *PTPN11*, *GLIS3*, and *MORF4L1* [21]. The enrichment of Alu repeats within T1D genes suggests their regulatory role in controlling expression of these genes. Some of the highly significant T1D-associated SNPs [28] were found to be present within the Alu elements of T1D genes. Specifically, 14 T1D genome-wide association study (GWAS) SNPs (*p*-value < 0.01) were found to be present within Alu elements of 16 T1D genes (see Supplementary Table S2).

We next compared the frequency of Alus within the coding, intronic, and UTR sequences of T1D genes (Table 2). The majority of the Alu elements in T1D genes were found to reside within the intronic sequences, followed by 3'UTRs, 5'UTRs, and coding regions with ~17%, ~4%, ~2%, and ~0.2% sequence coverage, respectively. Almost 80% of T1D intronic sequences harbored Alu elements, whereas only 1.77% of T1D coding sequences harbored Alu elements. The Alu elements accounted for almost 3% and 8% sequence coverage in T1D 5' and 3'UTRs, respectively. We further focused on the Alu distribution within T1D UTRs.

**Table 2:** Distribution of Alu repeats in T1D genes. The distribution of Alu repeats within T1D genes, T1D coding sequences (CDS), T1D intronic sequences, and T1D UTRs.

Category	T1D genes	CDS	Introns	5'UTRs	3'UTRs
Total number of repeats	37,101	660	125,952	582	1025
Total number of Alus	11,335	56	40,990	68	217
Sequences harboring repeats	81.08%	20.95%	92.09%	20.41%	30.48%
Sequences harboring Alus	59.29%	1.77%	80.44%	3%	8.02%
One Alu element occurrence per	1771 nt (1.7 kb)	45,868 nt (45 kb)	1580 nt (1.5 kb)	7234 nt (7.2 kb)	5933 nt (5.9 kb)
Percentage sequence covered by Alus	15.03%	0.17%	16.53%	1.83%	3.88%

### 3.2. Embedded Alu Elements within T1D UTRs

Since embedded Alus have been found to be enriched within the UTRs, we next compared the overall repeat content of background UTRs with T1D UTRs. The total percentage of sequence covered by repeats was 9.6% and 11.8% in background 5' and 3'UTRs, respectively. The background UTR dataset is described in Supplementary Table S3. Interspersed repeats collectively accounted for 6.3% and 10.6% sequence coverage in 5' and 3'UTRs, respectively (Supplementary Table S4). The most abundant interspersed repeat family in the background UTR dataset was SINES, corresponding to around 3.2% in 5'UTRs and 5.1% in 3'UTRs (Supplementary Table S4). In the case of both 5' and 3'UTRs, our results indicated over-representation of Alu repeat elements, with Alus alone accounting for 2% and 4% sequence coverage in 5' and 3'UTRs, respectively (see Supplementary Table S5). Moreover, out of 80,296 5'UTRs, only 2400 (3%) harbored Alu elements, whereas out of 72,248 3'UTRs, 8029 (11.1%) harbored Alu elements. Due to the intrinsic differences in length of 5'UTRs and 3'UTR, we also calculated the number of Alus per base. We found occurrence of one Alu element per 7129 and 5735 bases in 5' and 3'UTRs, respectively. Introduction of any bias due to differences in length of 5' and 3'UTRs was circumvented by expressing the number of Alus per base. These results show that Alu elements are four times more abundant in 3'UTRs than 5'UTRs. Moreover, taking the orientation of Alu elements in 5' and 3'UTRs into account, we did not observe any significant difference in the number of antisense (49%) and sense (51%) elements in 3'UTRs (5867 antisense and 6075 sense Alu elements). In contrast, in 5'UTRs, we observed at least 2.5-fold differences in the number of antisense (75%) and sense (25%) elements (2028 antisense and 681 sense Alu elements).

**Table 3:** Top 20 T1D genes based on total number of repeats and Alu elements. The top 20 T1D candidate genes based on total number of repeats, SINE elements, and Alu repeats based on RepeatMasker v. 4 [23].

T1D Gene	Total Repeats	SINEs	Alu Elements
*RAD51B*	1619	528	278
*AFF3*	925	327	212
*RPH3A*	861	319	127
*GLIS3*	826	221	102
*CUX2*	805	387	221
*DOK6*	664	153	100
*BACH2*	589	178	98
*SKAP2*	515	195	137
*HECTD4*	488	260	230
*CLEC16A*	468	201	121
*CTD-3088G3.8*	427	212	151
*FBXL20*	414	274	267
*ATXN2*	358	210	196
*CFDP1*	351	174	158
*PTPN2*	290	144	125
*MTMR3*	273	135	118
*RP11-57A19.4*	255	155	142
*PTPN11*	248	140	128
*CDK12*	233	165	155
*MORF4L1*	219	135	116

However, in the case of T1D 5'UTRs, we observed over-representation of SINE elements compared to the background (all 5'UTRs) (see Supplementary Tables S1 and S4). The SINE elements accounted for 3.6% and 4.8% sequence coverage in T1D 5'UTRs and T1D 3'UTRs, respectively, thus suggesting no over-representation or enrichment of SINE elements within 3'UTRs compared to all background 3'UTRs. The Alu repeats covered approximately 1.8% and 3.8% sequence in T1D 5'UTRs and T1D 3'UTRs, respectively. While expressing the number of Alus per base in T1D UTRs, we observed similar results as reported above for the background UTRs (one Alu element per 7234 and 5933 bases in T1D 5' and 3'UTRs, respectively), thus suggesting again a preference for the accumulation of Alu elements in the 3'UTRs (Table 2). Taking the orientation of Alus into account, we found 75% antisense and 25% sense Alu elements for T1D 5'UTRs (51 antisense and 17 sense Alu elements). In the case of T1D 3'UTRs, we observed 48.3% antisense and 51.6% sense Alu elements (105 antisense and 112 sense Alu elements). It has been suggested that the insertion of Alus in a particular orientation does not occur randomly [29]; instead, the mRNAs harboring sense, antisense, or both Alus might mediate regulatory processes via these elements. Additionally, it has been shown that Alu elements can serve as donors of miRNA binding sites in the 3'UTRs of various genes [8,9,10]. Therefore, it can be envisaged that some of the T1D genes might also be under Alu-mediated miRNA regulation, which would be interesting to explore in future studies.

### 3.3. Inverted Alu Repeats (IRAlus) in T1D 3'UTRs

**Figure 2:**
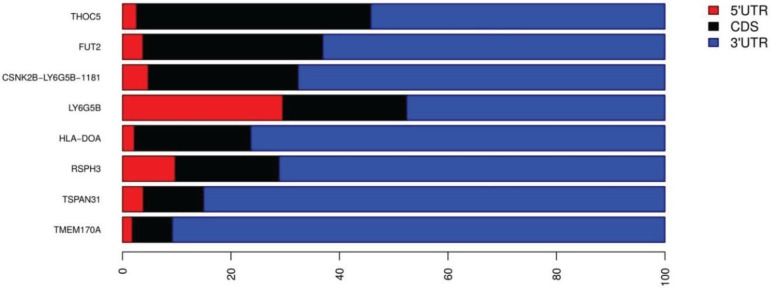
Contribution of UTRs and CDS to the length of T1D mRNAs harboring IRAlus. The contribution of 5'UTR, coding (CDS), and 3'UTRs to the total length is shown for eight T1D genes harboring IRAlus within their 3'UTRs. Only the protein-coding isoforms listed in Table 4 are shown. Transcripts are ranked according to the percentage of mRNA contributed by CDS. For genes with more than one protein-coding isoform, only the major isoform is shown. The x-axis represents the percentage of sequence coverage for each gene on the y-axis.

**Figure 3:**
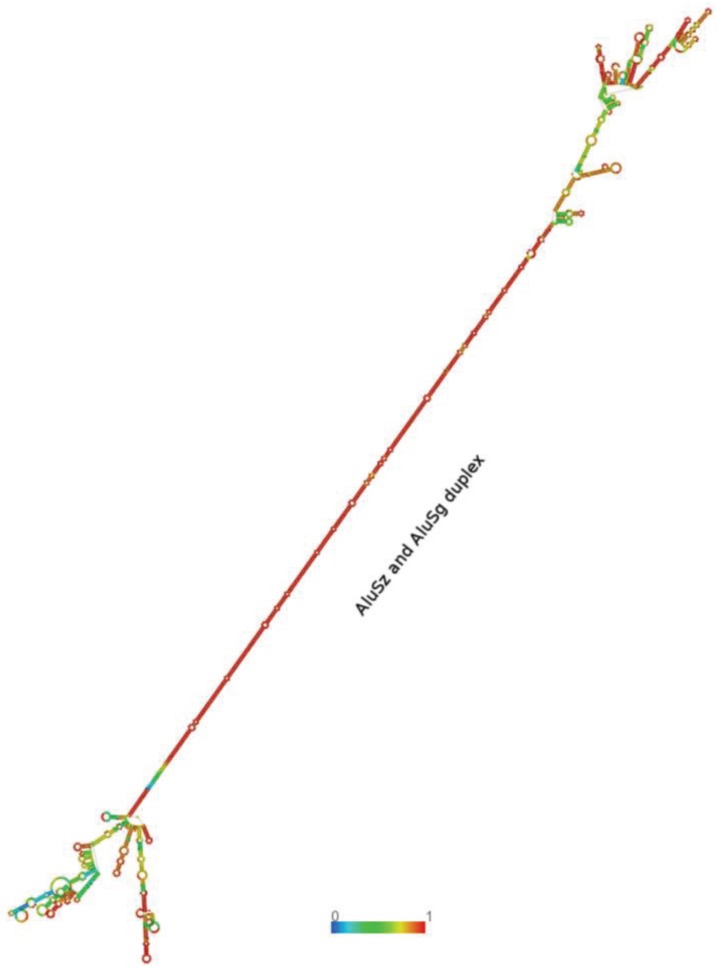
IRAlus within the 3'UTR of *FUT2*. The dsRNA duplex formed within the 3'UTR of *FUT2* (transcript id ENST00000425340) by intermolecular base-pairing between sense AluSz (311 nt) and antisense AluSg (307 nt) elements. The secondary structure was predicted by RNAfold. The color scale represents base-paring probability with values ranging from 0 to 1 and red color indicating strong base-pair probability.

The interaction between two Alus that are in opposite orientation (IRAlus) gives rise to dsRNAs duplexes, which in turn are known to decrease the translational efficiency [14]. IRAlus are known to undergo A-to-I editing at multiple sites by ADAR proteins [30,31]. Moreover, IRAlus are also known to be involved in downregulation of gene expression [32] and suppress apoptosis [33]. The T1D genes harboring IRAlus might also be involved in similar processes. We identified eleven T1D genes with IRAlus within their 3'UTRs (Table 4). However, some of the isoforms harboring IRAlus undergo nonsense-mediated decay (Table 4). For the protein-coding isoforms of eight T1D genes harboring IRAlus, we show that the length of their UTRs exceeds that of the coding region (Figure 2). This further emphasizes the importance of this region and suggests the extensive regulatory potential of UTRs in these genes. The T1D genes harboring IRAlus included some of the well characterized T1D susceptibility genes such as *HLA-DOA*, *FUT2*, and *SUOX* (Table 4). We predicted the secondary structure of 3'UTR of *FUT2* using RNAfold and RNAcofold in the ViennRNA package [25], which confirmed the dsRNA duplex formation by the embedded IRAlus (sense AluSz and antisense AluSg) (Figure 3). The estimated free energy of the duplex was found to be −513.34 kcal/mol. The free energy of IRAlus in isolation was −108.51 kcal/mol for sense AluSz and −105.73 kcal/mol for antisense AluSg. The duplex formation by IRAlus within the 3'UTRs of all eight T1D protein-coding mRNA transcripts was also confirmed by predicting secondary structures using RNAfold (see Supplementary Figure S1). The dsRNA structures formed by these IRAlus might be important regulators of the host gene expression.

**Table 4:** IRAlus within the 3'UTRs of T1D genes. The transcripts harboring IRAlus within 3'UTRs are listed with gene name, Ensembl transcript ID, total number of Alus, Alu subfamilies, length of the Alu repeat, and orientation of the Alu element (sense as “s,” and antisense as “a”). Some of the genes have more than one transcript with IRAlus.

Gene Name	Transcript ID	Total Alus	Alu subfamily	Alu Length	Alu Direction	Transcript Biotype
*CEP76*	ENST00000593250	6	AluSx, AluSx, AluSp, AluSp, Alu, AluSz6	149; 292; 293; 303; 49; 115	a;s;s;s;s;s	Nonsense-mediated decay
*TSPAN31*	ENST00000547992	5	AluSx, AluSx, FLAM_C, AluSc, FRAM	296; 310; 111; 289; 198	a;a;s;s;s	Protein coding
*THOC5*	ENST00000490103	4	AluSz, AluJr, AluJo, AluSx	297; 303; 296; 275	s;s;a;s	Protein coding
*RSPH3*	ENST00000367069	4	AluSp, AluJb, AluSc, AluSc	306; 315; 291; 284	s;a;s;s	Protein coding
*TMEM170A*	ENST00000357613	4	AluSg4, AluJb, AluY, AluSx1	303; 272; 288; 300	a;s;a;a	Protein coding
*TMEM170A*	ENST00000568559	4	AluSg4, AluJb, AluY, AluSx1	216; 241; 288; 308	a;s;a;a	Nonsense-mediated decay
*FUT2*	ENST00000425340	3	AluSz, AluSg, FAM	311; 307; 156	s;a;s	Protein coding
*CTD-3088G3.8*	ENST00000595170	3	AluSz6, AluSg, AluSx1	100; 75; 311	s;a;s	Nonsense-mediated decay
*HLA-DOA*	ENST00000229829	2	AluJr, AluSc	313; 283	s;a	Protein coding
*CSNK2B- LY6G5B-1181*	ENST00000409691	2	AluSx3, AluSz	292; 290	a;s	Protein coding
*CSNK2B- LY6G5B-1181*	ENST00000375880	2	AluSx3, AluSz	292; 290	a;s	Protein coding (Major isoform)
*LY6G5B*	ENST00000375864	2	AluSx3, AluSz	292; 290	a;s	Protein coding
*SUOX*	ENST00000550065	2	AluSq, AluSx	216; 200	s;a	Nonsense-mediated decay

### 3.4. T1D Genes Harboring Alu Repeats Are Enriched in Immune-Mediated Processes

For the 554 T1D genes harboring Alu repeats, we performed functional annotation analysis based on gene ontology (GO) terms and pathways using DAVID [26]. We searched for enriched functional categories using the GO-FAT classification, as the GO-FAT category is more specific and filters out the broadest terms in hierarchy. The GO-based analysis revealed significantly enriched GO terms in biological process and cellular component categories (Table 5). The T1D genes harboring Alus were found to be enriched for immune-mediated processes including antigen processing and presentation (*p*-value = 2.12e−14), immune response (*p*-value = 5.77e−13), and defense response (*p*-value = 8.29e−10) in BP-FAT category. In the cellular component category, top enriched processes included MHC protein complex (*p*-value = 3.07e−11) and plasma membrane part (*p*-value = 1.34e−07). Nine pathways were found to be enriched for T1D genes harboring Alu repeats at a significance level of 0.05 (Table 6), based on KEGG pathway categories [27]. The top enriched pathway included Allograft rejection (*p*-value = 5.40e−10), Type 1 diabetes mellitus (*p*-value = 3.39e−09), and antigen processing and presentation (*p*-value = 1.10e−08). Table 6 lists all the enriched pathways for T1D genes harboring Alu repeats. Similar annotations were found enriched for T1D genes harboring Alu repeats based on functional annotation clustering by DAVID [26] (data not shown).

**Table 5:** GO term-based annotation of T1D genes harboring Alu elements. The enriched GO terms are followed by the number of genes having the enriched term (count), the percentage of genes with the enriched term (%), *p*-values based on EASE scores, and Bonferroni correction *p*-values.

Term		Count	%	*p*-Value	Bonferroni
Biological Process (BP_FAT)					
1	antigen processing and presentation	19	5.22	2.12e−14	3.98e−11
2	immune response	46	12.64	5.77e−13	1.08e−09
3	defense response	38	10.44	8.29e−10	1.56e−06
4	positive regulation of immune system process	22	6.04	7.71e−09	1.45e−05
5	positive regulation of immune response	16	4.40	1.43e−07	2.69e−04
6	positive regulation of response to stimulus	19	5.22	8.25e−07	1.55e−03
7	response to unfolded protein	10	2.75	9.00e−06	0.02
8	regulation of T cell activation	12	3.30	1.71e−05	0.03
9	inflammatory response	20	5.49	2.00e−05	0.04
10	regulation of leukocyte activation	14	3.85	2.16e−05	0.04
Cellular Component (CC_FAT)					
1	MHC protein complex	14	3.85	3.07e−11	9.33e−09
2	plasma membrane part	75	20.60	1.34e−07	4.06e−05
3	integral to plasma membrane	42	11.54	9.18e−05	0.03
4	intrinsic to plasma membrane	42	11.54	1.50e−04	0.04

**Table 6:** KEGG pathway-based annotation of T1D genes harboring Alu elements. The enriched pathway names are followed by the number of genes within the enriched pathway (count), the percentage of genes with the enriched pathway (%), *p*-values based on EASE scores, and Bonferroni correction.

KEGG PATHWAY		Count	%	*p*-value	Bonferroni
1	Allograft rejection	12	3.30	5.40e−10	7.29e−08
2	Type 1 diabetes mellitus	12	3.30	3.39e−09	4.58e−07
3	Antigen processing and presentation	15	4.12	1.10e−08	1.48e−06
4	Graft-versus-host disease	11	3.02	2.28e−08	3.08e−06
5	Autoimmune thyroid disease	12	3.30	3.12e−08	4.21e−06
6	Viral myocarditis	13	3.57	1.28e−07	1.73e−05
7	Intestinal immune network for IgA production	11	3.02	2.38e−07	3.22e−05
8	Cell adhesion molecules (CAMs)	15	4.12	4.03e−06	5.44e−04
9	Systemic lupus erythematosus	13	3.57	5.11e−06	6.89e−04

## 4. Conclusions

In conclusion, our analysis highlights the importance of Alu repeat elements within T1D genes. Given the involvement of Alu elements in various cellular processes including regulation of gene expression, it is plausible to envision similar roles played by both sense and antisense Alu elements within the T1D genes. In addition, the enrichment of immune-mediated processes in our pathway and functional annotation analysis further indicates the potential functional roles of Alu elements in T1D pathogenesis. Taken together, our findings suggest that Alu harboring transcripts encompass a novel class of gene expression regulators in the T1D context. Nevertheless, findings from this study necessitate a systematic experimental follow-up using relevant Type 1 diabetes cellular and murine models. Notably, since many interspersed repeat elements are known to be enriched in regulatory regions, including enhancers, it would therefore be interesting to investigate the role of embedded enhancer-associated Alus identified within the 5' and 3'UTRs of T1D genes. In particular, identification of Alu elements that are transcribed and whose expression is modulated by pro-inflammatory cytokines treatment in insulin producing beta-cells would be an important step to understand their role in T1D pathogenesis.

## Supplementary Files

Supplementary File 1
