# Supplementary material for: Alu Elements as Novel Regulators of Gene Expression in Type 1 Diabetes Susceptibility Genes?
Source: Genes (Basel). 2015 Jul 13;6(3):577–91. doi: 10.3390/genes6030577 (PMC4584318; doi:10.3390/genes6030577)
Supplement: Supplementary File 1 [file genes-06-00577-s001.pdf]

Supplementary Tables

Table S1. Percentage sequence covered by repeats for sequences including coding (CDS) sequences, intronic sequences and UTRs (untranslated regions) of T1D genes.

	**T1D**				
**Repeat Class**	**T1D Genes**	**CDS**	**Introns**	**5'UTRs**	**3'UTRs**
SINEs	18.07%	0.19%	19.88%	3.66%	4.87%
LINEs	15.44%	0.13%	16.08%	1.17%	2.17%
LTRs	4.85%	0.04%	4.29%	0.46%	1.74%
DNA elements	3.29%	0.03%	3.74%	0.35%	1.14%
Unclassified	0.24%	0%	0.33%	0.05%	0%
Small RNA	0.08%	0%	0.05%	0%	0%
Satellites	0.03%	0%	0.01%	0%	0%
Simple repeats	1.10%	0.84%	1.10%	2.91%	1.17%
Low complexity regions	0.19%	0.17%	0.16%	0.40%	0.19%
Total percentage sequence covered by repeats	43.30%	1.40%	45.64%	9%	11.28%

Table S2. T1D associated SNPs within Alu elements of T1D genes. The T1D GWAS SNPs were retrieved from Barrett *et al.*, 2009 with a *p*-value cutoff < 0.01. The T1D genes with highly significant GWAS *p*-values are highlighted in red. The orientation of the Alu elements is depicted with + for sense Alus and C for antisense Alus.

Chr	SNP Position	SNP	Alu Start	Alu End	ALU Length	Alu Direction	Alu Subfamily	Ensembl ID	Gene Name	Strand	GENE TYPE	*p*-Value (Barrett *et al.*, 2009[1])
chr10	6192811	rs11256997	6192679	6192988	310	+	AluSx1	ENSG00000170525	PFKFB3	1	protein coding	1.70E-03
chr10	6471816	rs11596750	6471681	6471904	224	+	AluJb	ENSG00000065675	PRKCQ	−1	protein coding	2.00E-06
chr19	10537268	rs1352426	10537265	10537560	296	+	AluSz6	ENSG00000065989	PDE4A	1	protein coding	6.80E-03
chr16	11293711	rs248848	11293652	11293937	286	C	AluSg7	ENSG00000263033	RP11-396B14.2	1	lincRNA	1.20E-03
chr7	26908972	rs213527	26908970	26909256	287	C	AluSx	ENSG00000005020	SKAP2	−1	protein coding	2.00E-06
chr6	29971548	rs6905157	29971299	29971586	288	+	AluJb	ENSG00000204623	ZNRD1-AS1	−1	antisense	1.50E-10
chr6	30302600	rs3778624	30302514	30302799	286	C	AluJr4	ENSG00000204599	TRIM39	1	protein coding	2.40E-04
chr6	30302600	rs3778624	30302514	30302799	286	C	AluJr4	ENSG00000248167	TRIM39-RPP21	1	protein coding	2.40E-04
chr6	30434900	rs7747873	30434643	30434912	270	C	AluSz	ENSG00000229068	TMPOP1	1	pseudogene	3.10E-07
chr6	30735105	rs12210947	30734802	30735111	310	C	AluJb	ENSG00000228022	HCG20	1	lincRNA	5.60E-39
chr6	30913458	rs3132581	30913450	30913752	303	+	AluSc8	ENSG00000168631	DPCR1	1	protein coding	2.40E-85
chr6	31091992	rs3094204	31091985	31092027	43	+	FAM	ENSG00000204540	PSORS1C1	1	protein coding	5.20E-03
chr6	31673436	rs9267546	31673135	31673443	309	+	AluSc	ENSG00000204422	XXbac-BPG32J3.20	−1	protein coding	1.20E-27
chr6	33150268	rs7382464	33150207	33150501	295	C	AluSz	ENSG00000204248	COL11A2	−1	protein coding	1.30E-10
chr12	56564811	rs7960225	56564509	56564814	306	+	AluSg	ENSG00000139613	SMARCC2	−1	protein coding	1.20E-21
chr12	56564811	rs7960225	56564509	56564814	306	+	AluSg	ENSG00000258199	RP11-977G19.5	−1	sense overlapping	1.20E-21

Table S3. Background UTR dataset. Characteristics of human transcriptome-wide UTRs based on Ensembl v75 (GrCh37).

All UTRs			
**Category**	**5'UTRs**	**3'UTRs**	**Total**
Total UTRs	80,296	72,248	
Total Genes	19,574	19,639	19,969
Average UTR per gene	4.1	3.67	
Genes with single transcripts	4968	4930	
Genes having both 5' and 3' UTRs			19,244
Average length of UTRs	240	948	
Max length of UTRs	14,960	22,552	

Table S4. Percentage of sequence covered by repeats in background UTR dataset.

All UTRs		
**Repeat Class**	**5'UTRs**	**3'UTRs**
SINEs	3.20%	5.14%
LINEs	1.50%	2.63%
LTRs	1.04%	1.49%
DNA elements	0.55%	1.33%
Unclassified	0.03%	0.04%
Small RNA	0.01%	0.03%
Satellites	0.05%	0.01%
Simple repeats	2.69%	1%
Low complexity regions	0.51%	0.18%
Total percentage of sequence covered by repeats	9.58%	11.85%

Table S5. Distribution of Alu repeats in the background UTR dataset.

All UTRs		
**Category**	**5'UTRs**	**3'UTRs**
Total UTRs	80,296	72,248
Total bases in UTRs	19,314,465	68,496,749
Total number of UTRs having repeats	16,390	25,840
Total number of UTRs having Alu's	2400	8029
Total number of UTRs having SINEs	3969	11,104
Total number of repeats in UTRs	21,679	55,353
Total number of Alu's in UTRs	2709	11,942
Total number of SINEs in UTRs	4669	17,669
Percentage of UTRs having repeats	20.41%	35.76%
Percentage of UTRs having Alu's	3%	11.11%
Percentage of UTRs having SINEs	4.94%	15.36%
One Alu element occurrence per	7129 bases (7.1 kb)	5735 bases (5.7kb)
Percentage sequence covered by Alus	2.08%	4.07%
Percentage sequence covered by SINEs	3.20%	5.14%

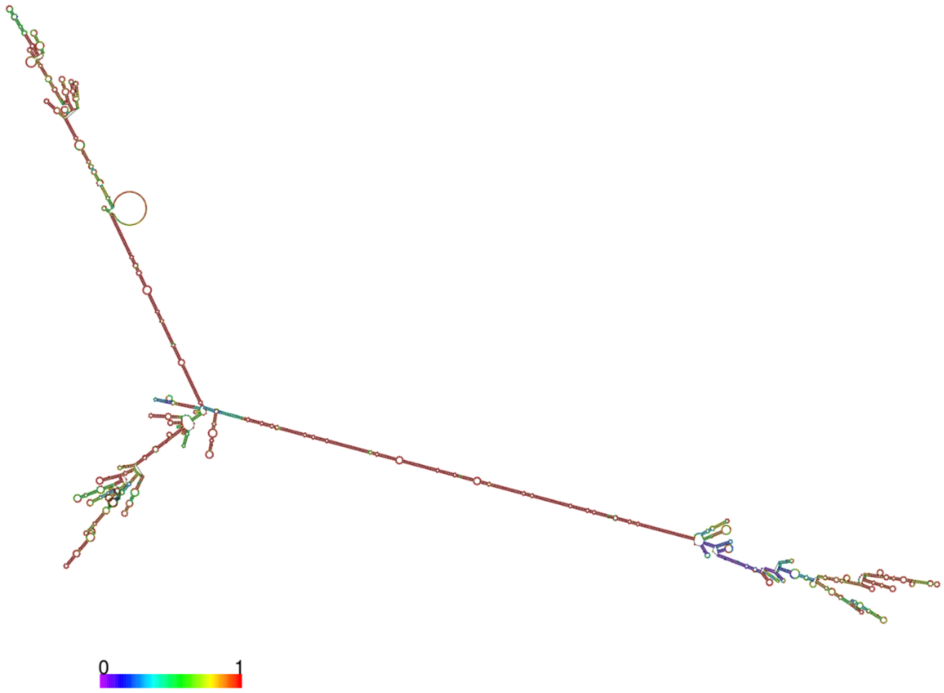

(**A**)

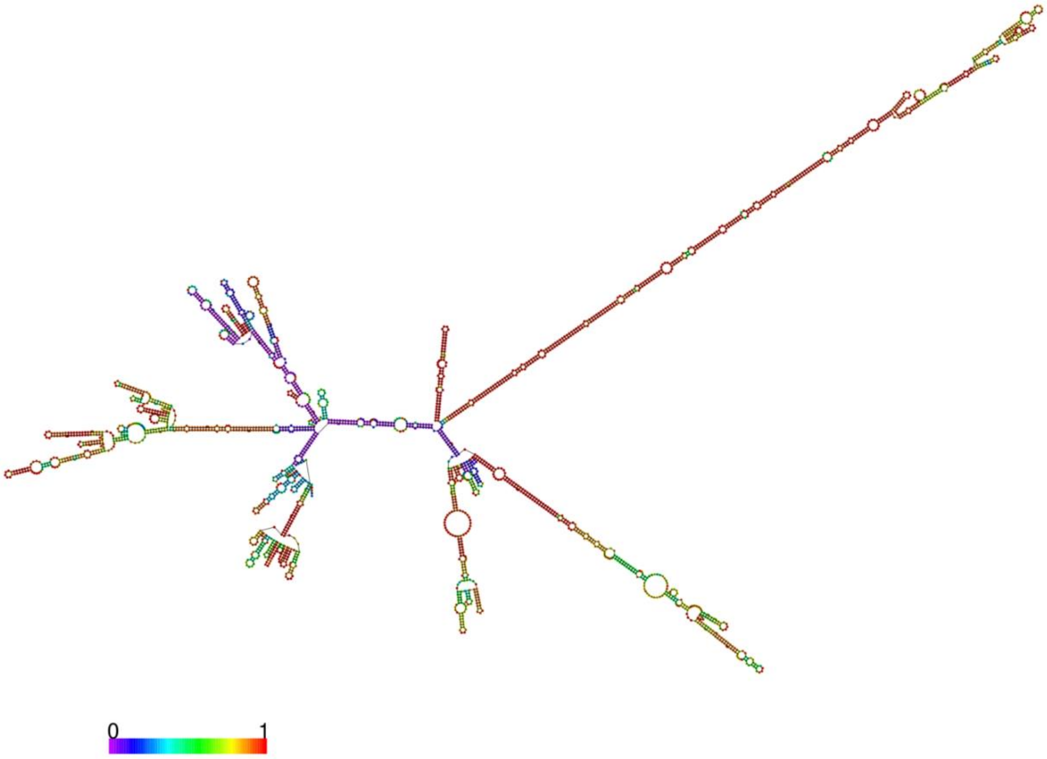

(**B**)

Figure S1. *Cont.*

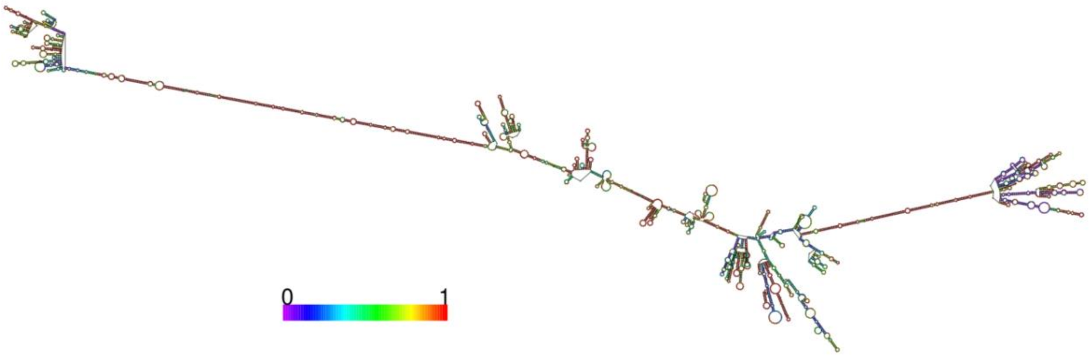

(**C**)

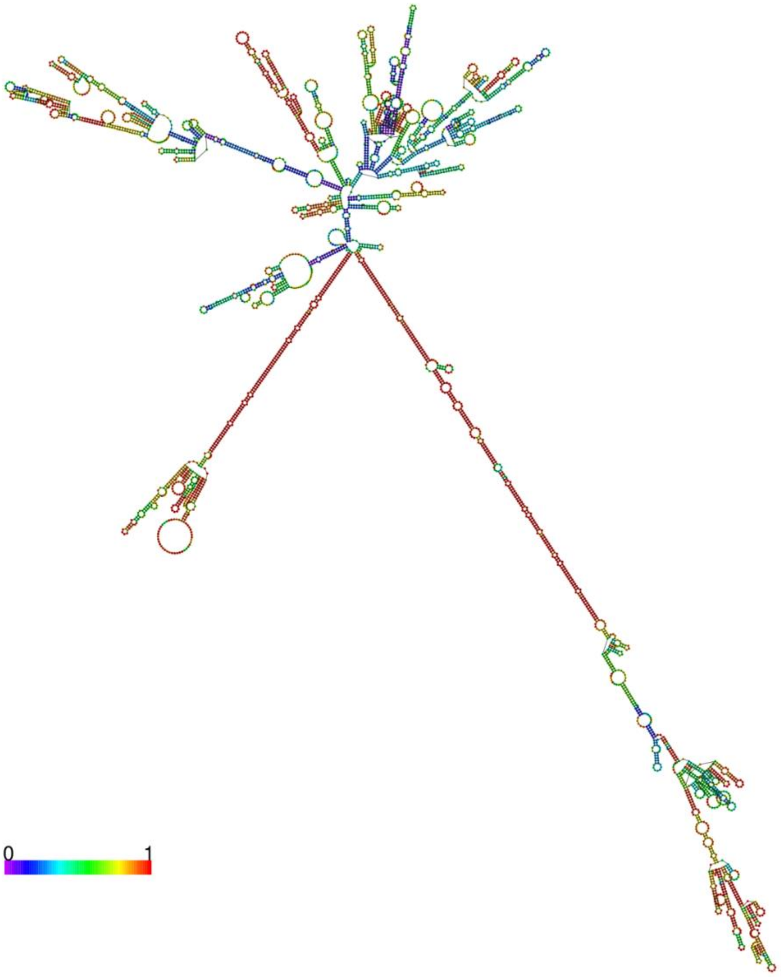

(**D**)

Figure S1. *Cont.*

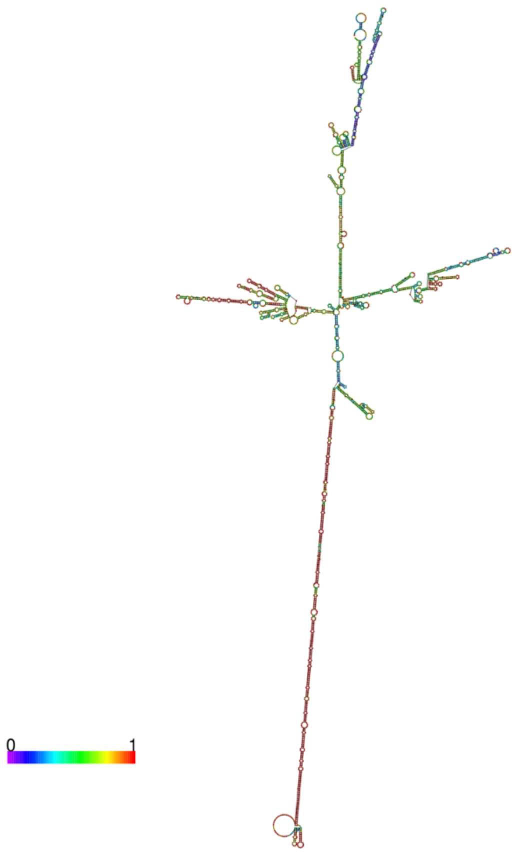

(**E**)

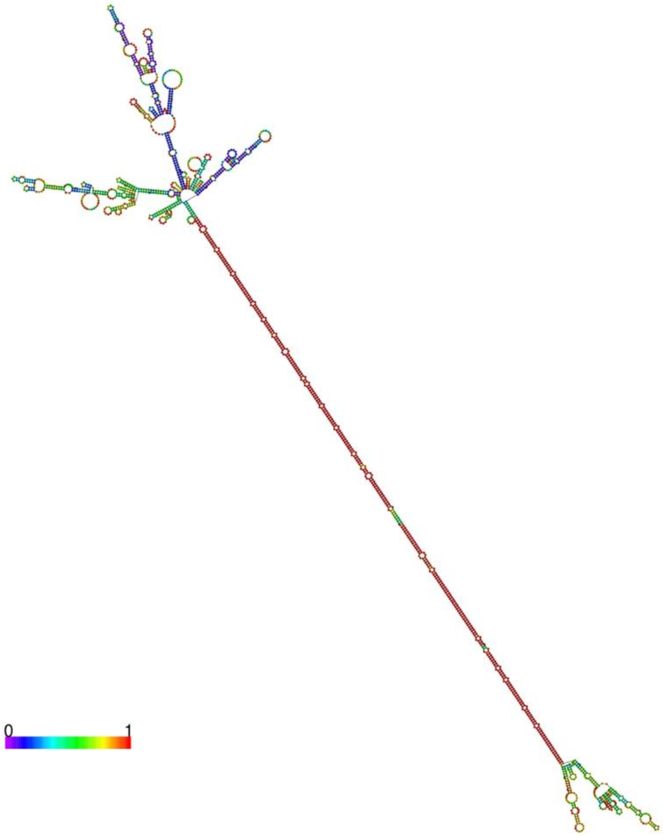

(**F**)

Figure S1. *Cont.*

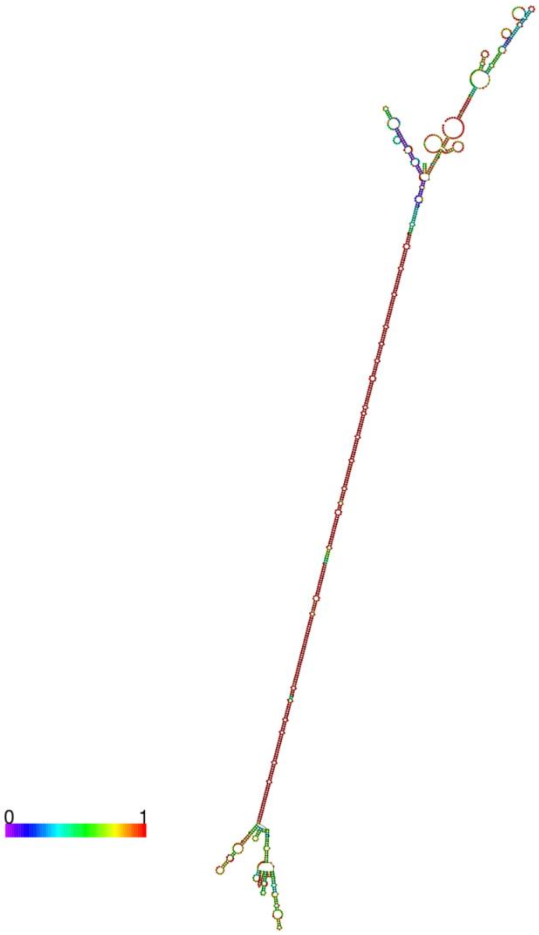

(**G**)

Figure S1. The dsRNA duplex formed by IRAlus within 3’UTRs of T1D mRNAs. The secondary structures were predicted by RNAfold. The color scale represents base-paring probability with values ranging from 0 to 1 and red color indicating strong base-pair probability. (**A**) Secondary structure of *TSPAN31* 3’UTR (ENST00000547992); (**B**) Secondary structure of *THOC5* 3'UTR (ENST00000490103); (**C**) Secondary structure of *RSPH3* 3'UTR (ENST00000367069); (**D**) Secondary structure of *TMEM170A* 3'UTR (ENST00000357613); (**E**) Secondary structure of *HLA-DOA* 3'UTR (ENST00000229829); (**F**) Secondary structure of *CSNK2B-LY6G5B-1181* 3'UTR (ENST00000375880); (**G**) Secondary structure of *LY6G5B* 3'UTR (ENST00000375864).

Reference

1. Barrett, J.C.; Clayton, D.G.; Concannon, P.; Akolkar, B.; Cooper, J.D.; Erlich, H.A.; Julier, C.; Morahan, G.; Nerup, J.; Nierras, C.; *et al*. Genome-wide association study and meta-analysis find that over 40 loci affect risk of type 1 diabetes. *Nat. Genet.* **2009**, *41*, 703–707.
